# Inflammatory Complications in Chronic Granulomatous Disease

**DOI:** 10.3390/jcm13041092

**Published:** 2024-02-15

**Authors:** Alexandros Grammatikos, Andrew R. Gennery

**Affiliations:** 1 The Bristol Immunology and Allergy Centre, North Bristol NHS Trust, Bristol BS10 5NB, UK; 2 Paediatric Stem Cell Transplant Unit, Great North Children’s Hospital, Newcastle upon Tyne NE1 4LP, UK; andrew.gennery@newcastle.ac.uk

**Keywords:** chronic granulomatous disease, inflammatory complications, granulomatous inflammation, X-linked, autosomal recessive, colitis, bowel obstruction, interstitial lung disease, cystitis

## Abstract

Chronic granulomatous disease (CGD) is a rare inborn error of immunity that typically manifests with infectious complications. As the name suggest though, inflammatory complications are also common, often affecting the gastrointestinal, respiratory, urinary tracts and other tissues. These can be seen in all various types of CGD, from X-linked and autosomal recessive to X-linked carriers. The pathogenetic mechanisms underlying these complications are not well understood, but are likely multi-factorial and reflect the body’s attempt to control infections. The different levels of neutrophil residual oxidase activity are thought to contribute to the large phenotypic variations. Immunosuppressive agents have traditionally been used to treat these complications, but their use is hindered by the fact that CGD patients are predisposed to infection. Novel therapeutic agents, like anti-TNFa monoclonal antibodies, anakinra, ustekinumab, and vedolizumab offer promise for the future, while hematopoietic stem cell transplantation should also be considered in these patients.

## 1. Introduction

Chronic granulomatous disease (CGD) is a rare inborn error of immunity caused by a genetic defect in one of the components of nicotinamide adenine dinucleotide phosphate (NADPH) oxidase complex. The latter is required for the generation of reactive oxygen species, which are essential to kill phagocytosed pathogens. As a consequence, neutrophils in these patients are unable to kill phagocytosed pathogens and prevent the spread of infections. NADPH oxidase is composed of 2 membrane proteins (gp91phox and p22phox) and 4 cytosolic proteins (p47phox, p67phox, p40phox, and Rac1/2). Both membrane-bound components are required to stabilise their counterpart expression, so when either one is missing, the other is not expressed.

CGD can be inherited in an X-linked (XL) or an autosomal recessive (AR) manner. Six genes are involved, namely CYBB (XL), encoding for gp91phox protein, NCF1 (AR), encoding for p47phox protein, NCF2 (AR), encoding for p67phox protein, NCF4 encoding for p40phox, and CYBA (AR), encoding for p22phox protein [1]. Stabilisation of the assembled cytochrome in the endoplasmic reticulum membrane is achieved by the protein, ‘essential for reactive oxygen species’ (EROS), encoded by CYBC1, defects of which can also cause CGD [2]. In countries where non-consanguineous unions are prevalent, XL-CGD is more common than AR-CGD.

Diagnosis is usually made early in life using a flow cytometric oxidative (respiratory) burst assay. These assays utilise either dihydrorhodamine 123 (DHR) or nitroblue tetrazolium (NBT) to detect oxygen radical production levels by neutrophils. More definite diagnosis is achieved by genetic testing, which can also provide information on the inheritance mode of the disorder.

The disease typically manifests early in life with recurrent abscesses, pneumonias, lymphadenitis, or osteomyelitis by catalase-positive bacteria (e.g., Staphylococcus aureus) and fungi (e.g., Aspergillus fumigatus). Patients with the XL form of the disease tend to be diagnosed earlier in life than those with AR. Patients also frequently exhibit inflammatory complications, with the histological hallmark being granulomatous tissue [3,4]. Inflammation is frequently overlooked, and often co-exists with infection. Infections tend to precede inflammatory manifestations, but in one series, most patients had their first inflammatory episode by the age of 20 [5]. Although female carriers of CYBB mutations (XL) are not usually troubled by infections, they often exhibit inflammatory complications.

The severity of the pathogenic defect seems to be associated to the variable clinical phenotype [6]; Kuhns et al., first reported that absent neutrophil residual oxidase activity (NROA) is associated with a higher risk of infection and more severe illness [7], with severity associated to the reduction or absence of phagocyte-derived superoxide and related reactive oxygen intermediates, rather than the NADPH subunit. The different genetic mutations that lead to AR-CGD are linked to different NROA. E.g., NROA in p47phox deficiency, the most common type of AR-CGD, is significantly higher than that in p22phox and p67phox deficiencies, and likely leads to the erroneous conclusion that AR-CGD is less severe than XL-CGD. In reality, infection and inflammatory complications in patients with loss-of-function mutations in the latter two sub-types confer similar disease severity as that experienced by XL-CGD patients, as the NROA in the latter two is comparable [8,9,10,11]. 

Nonspecific chronic inflammation is a common histopathologic finding in these patients, and fibrotic tissue containing noncaseous granulomas are also frequently seen. Complications can be caused by either microscopic or macroscopic granulomas, the latter causing mechanical obstruction, e.g., of the digestive or urinary tract [12]. Histologically, granulomas consist of a spherical structure with a central core of tissue resident macrophages (which may merge into multinucleated giant cells) surrounded by T cells. They develop in response to an antigen, e.g., bacteria or fungi, and are thought to be an attempt of the immune system to control spread of these infections. Granulomas are, however, often also seen in autoimmune diseases and after exposure to certain substances like silica dust and beryllium. In CGD, gut biopsies reveal noncaseating granulomata, along with an eosinophil-rich infiltrate, crypt abscesses, and large pigment-containing macrophages in the lamina propria [5].

## 2. Prevalence and Clinical Manifestations

Sub-clinical inflammation is common, and often missed. Inflammatory complications are reported more frequently in XL-CGD patients, although that may be an ascertainment bias. In a French study, an average of 0.15 inflammatory episodes per person-year were reported, with the relative risk being significantly greater than in AR-CGD (RR = 2.22) [5]. Formation of granulomata and dysregulated inflammation in CGD contribute to morbidity and can cause multiple symptoms. The genitourinary and gastrointestinal tracts are most commonly affected.

Gastrointestinal inflammatory complications tend to be most common. Symptomatic inflammatory bowel disease (IBD) affects up to 61% of individuals and can be the presenting finding (Table 1) [13]. Marciano et al. found that XL-CGD patients exhibit a significantly larger number of gastrointestinal inflammatory complications compared to AR-CGD [13]. Symptoms can vary depending on the site affected and include abdominal pain, diarrhoea, constipation, weight loss, fever, nausea, and rectal bleeding. Oesophageal, jejunal, ileal, caecal, rectal, and perirectal granulomata similar to those seen in Crohn’s disease are described [14,15]. Significant colitis leading to bowel obstruction, fistulae, and strictures can result in growth restriction [13]. Pyloric oedema leads to functional gastric outlet obstruction and can be an initial presentation of CGD [14,15]. Also, oesophagitis, oesophageal obstruction or stricture, oesophageal diverticulitis, and eosinophilic gastritis have been described [16]. Oral manifestations include gingivitis, stomatitis, aphthous ulceration, and gingival hypertrophy. CGD-associated hepatic disease is also a significant cause of morbidity. Portal venopathy can be associated with splenomegaly and nodular regenerative hyperplasia. Portal hypertension with thrombocytopenia is associated with intrahepatic disease and is a risk factor for mortality [17].

Non-infectious pulmonary complications occur in up to a third of patients with CGD. Patients with XL-CGD seem to have a higher risk of developing these than those with AR-CGD. Manifestations include interstitial lung disease, pulmonary nodules, and, less often, obliterative bronchiolitis, chronic fibrosis, and chronic obstructive pulmonary disease [18]. Lung granulomas can present with obstructive symptoms, depending on their location in the pulmonary tree. Radiologic findings are often non-specific and can include consolidation, ground-glass opacities, tree-in-bud opacities, scattered nodules, bronchiectasis, and even mimic neoplasms. A rare presentation is mulch pneumonitis, described in XL- and AR-CGD, and associated with invasive filamentous fungal infection and hyperinflammation. Patients present acutely unwell with hypoxia and require treatment with corticosteroids as well as antifungals [19].

Inflammatory complications of the urinary tract are reported in up to 19% of patients. (Table 1). Again, primarily large granulomas that lead to obstruction or stricture at various locations are reported (ureteral, urethral, etc.), but also eosinophilic cystitis, pseudotumors of the bladder, and urethritis [14,15]. Renal complications like glomerulonephritis and unexplained renal impairment/scarring have also been reported in previous case series [5,15].

Ophthalmic inflammatory complications include chorioretinal lesions and granulomata with pigment clumping that are usually asymptomatic [20]. Inflammatory eye disease including keratitis and uveitis can also occur. In a prospective study of 36 patients (31 XL-CGD and 5 AR-CGD), chorioretinal lesions were identified in 9 of them, which is much higher than what is reported by retrospective studies (Table 1).

Cutaneous granulomatous lesions can be seen in these patients. Granulomatous acne, inflammatory nodular lesions, photosensitivity, cutaneous lymphocytic infiltration, vasculitis and pyoderma gangrenosum have been described in association with CGD [15,21]. Poor wound healing with excessive inflammation at drainage and surgical wounds leading to dehiscence can also be seen.

Autoimmune disorders are also more common, including idiopathic thrombocytopenic purpura, juvenile idiopathic arthritis, autoimmune pulmonary disease, myasthenia gravis, IgA nephropathy, antiphospholipid syndrome, and recurrent pericardial effusion [14,22]. Substantially higher rates of systemic lupus erythematosus (SLE, 0.5% of patients) or discoid lupus (2.7% of patients) are seen in these patients. Autoimmunity is also common in carriers of XL-CGD, who may present with an SLE-like syndrome, although the classical autoantibodies associated with SLE are often not present.

Prolonged and dysregulated inflammation in CGD can even overlap clinically with the syndrome of hemophagocytic lymphohistiocytosis (HLH) [23]. Patients with CGD can develop prolonged fever and most of the clinical features of HLH.

## 3. Published Case Series

Several large series of CGD patients have been published and in most of them, the majority of cases were XL-CGD [5,14,15,21,24,25,26,27,28,29,30]. The most common inflammatory complications observed in these were gastrointestinal 34%, respiratory 13%, urinary 10%, cutaneous 9%, hepatic 5%, ocular 5%, connective tissue 1%, haematologic (cytopenias) 0.5%, and neuromuscular 0.3%, in a total number of 1311 patients (Table 1).

**Table 1:** Inflammatory complications reported in large case series with a majority of XL-CGD patients.

	Van den Berg et al., 2009 (Europe) [15]	Winkelstein et al., 2000 (USA) [14]	Bortoletto et al., 2015 (USA) [30]	Magnani et al., 2014 (France) [5]	Dunogue et al., 2017 (France) [21]	Jones et al., 2008 (UK) [29]	Cale et al., 2000 (UK) [28]	Oliveira-Junior et al., 2015 (Latin America) [27]	Martire et al., 2007 (Italy) [26]	Liese et al., 2020 (Germany) [25]	Raptaki et al., 2013 (Greece) [24]	Mean Frequency (range)
**Number of patients**	429 (290 XL, 139 AR)	368 (259 XL, 81 AR)	27 (19 XL, 8 AR)	98 (70 XL, 20 AR)	80 (59 XL, 21 AR)	94 (69 XL, 16 AR)	21 (14 XL, 4 AR)	71 (53 XL, 16 AR)	60 (39 XL, 6 AR)	39 (32 XL, 7 AR)	24 (16 XL, 7 AR)	
**Gastrointestinal**	58 (14%)	124 (34%)	11 (41%)	60 (61%)	43 (54%)	46 (49%)	10 (48%)	14 (20%)	5 (8%)	9 (23%)	5 (21%)	34% (8–61%)
Oesophageal ^1^	9	3	0	0	9	5	2	0	2	2	0	
Gastric ^2^	9	57	1	>10		6	0	0	0	2	3	
Colonic ^3^	40	64	10	>32	34	35	8	14	3	5	2	
**Respiratory ^6^**	0	0	1 (4%)	18 (18%)	25 (31%)	23 (25%)	0	24 (34%)	0	9 (23%)	1 (4%)	13% (0–34%)
**Urinary**	43 (10%)	37 (10%)	5 (19%)	12 (12%)	8 (10%)	13 (14%)	3 (14%)	4 (6%)	1 (2%)	2 (5%)	2 (8%)	10% (2–19%)
Renal ^4^	18	0	3	12	3	6	0	4	0	0	1	
Collective system ^5^	25	37	2		5	7	3		1	2	1	
**Cutaneous ^9^**	28 (7%)	10 (3%)	2 (8%)	10 (10%)	32 (40%)	0	2 (10%)	12 (17%)	3 (5%)	0	0	9% (0–40%)
**Hepatic ^8^**	1 (0.2%)	0	1 (4%)	0	10 (13%)	0	0	12 (17%)	0	7 (18%)	1 (4%)	5% (0–18%)
**Ocular ^7^**	8 (2%)	8 (2%)	4 (15%)	6 (6%)	7 (9%)	17 (18%)	0	0	1 (2%)	0	0	5% (0–18%)
**Connective tissue ^10^**	5 (1%)	2 (0.5%)	0	5 (5%)	3 (4%)	0	0	0	1 (2%)	0	0	1% (0–5%)
**Cytopenias ^11^**	1 (0.2%)	5 (1%)	0	0	2 (3%)	0	0	0	0	0	0	0.5% (0–3%)
**Neuromuscular ^12^**	0	1 (0.2%)	0	2 (2%)	1 (1%)	0	0	0	0	0	0	0.3% (0–2%)

^1^ oesophagitis, oesophageal obstruction or stricture, oesophageal diverticulitis, ^2^ gastritis, gastric outlet obstruction, granulomas, eosinophilic gastritis, ^3^ ulcerative/Crohn’s/eosinophilic/inflammatory colitis, chronic diarrhoea without an infectious cause, ^4^ nephritis, glomerulonephritis, renal impairment/scarring, ^5^ ureteral /urethral obstruction with hydronephrosis, inflammatory/eosinophilic cystitis, urethritis, ^6^ obliterative bronchiolitis, pulmonary fibrosis, diffuse interstitial pneumonia, granulomas, ^7^ chorioretinitis, keratitis, uveitis, scars, retinitis pigmentosa, episcleritis, ^8^ autoimmune hepatitis, granulomas, chronic elevation of liver enzymes, fibrosis, ^9^ granulomas, discoid lupus, chilblains, lupus-like lesions, erythema nodosum, vitiligo, leukocytoclastic vasculitis, inflammatory nodular lesions, pyoderma gangrenosum, ^10^ systemic lupus erythematosus, rheumatoid arthritis, vasculitis, dermatomyositis, sacroiliitis, antiphospholipid syndrome, ^11^ autoimmune thrombocytopenia, haemolytic anaemia, cold agglutinin disease, ^12^ acute demyelinating encephalitis, CNS granulomas, myasthenia gravis.

Only a few large case series, in whom the majority of patients were AR-CGD, have been published (Table 2). Respiratory inflammatory complications were more common in them (23%). Most cases in those series had, however, defects that are expected to have NROA levels comparable to those of XL-CGD (p22phox and p67phox); i.e., 68.1% in Rawat et al., (genetic defect identified in 59.7%) [9], 98% in Mortaz et al., (genetic defect identified in 88%) [10], and 80% in Koker et al. (genetic defect identified in 94%) [8]. The only case series where the majority of cases (64%) were expected to have a higher NROA compared to XL-CGD (genetic defect identified in 84%) [11] identified very few inflammatory complications, which is likely to be more representative of p47phox defects.

## 4. XL-CGD Carriers

Female carriers of CYBB mutations have 2 populations of neutrophils: one expressing the wild type CYBB and another expressing the mutated one. The cells expressing wild-type CYBB have normal superoxide production, whereas the diseased allele will confer reduced or absent superoxide production. Because there is no survival advantage to wild type-expressing phagocytes, lyonisation can lead to a spectrum of superoxide production, from very low to near normal [31]. Most carriers are infection free, but many exhibit inflammatory complications.

In two large case series of 94 and 162 carriers, 70% and 19% of them, respectively, exhibited such complications, including: discoid lupus, SLE, granulomatous colitis, and thyroid abnormalities [32,33]. Unlike infection, in neither series were inflammatory complications associated with low superoxide production, but symptoms were found across the spectrum of superoxide production. In one small case series chorioretinitis was observed in 10% of carrier females. Other relevant manifestations that have been reported in these patients include eczema, photosensitivity, IBD, colon polyposis, polyarthritis, recurrent aphthous ulcers, and Raynaud’s.

## 5. Aetiopathogenesis

The pathogenetic mechanisms underlying inflammatory complications in CGD are not well understood but are likely multi-factorial and reflect the body’s attempt to control infections. Several pathways are likely to contribute, and one or more may predominate in any clinical episode.

Infection likely precipitates most inflammatory episodes, and failure of clearance of phagocytosed material is likely an important inflammatory stimulus [34]. Phagocytes from patients with CGD, that lack functional NADPH oxidase accumulate at sites of infection but fail to clear microbial material or cellular debris, which leads to persistent cell activation and an exaggerated inflammatory response [35]. E.g., CGD patients can develop localized or disseminated granulomatous inflammation following BCG vaccination (BCG-itis) [34]. A French study reported that 7.5% of CGD patients suffer from post-infectious granulomatous inflammation in various organs [21]. The median age of the first inflammatory episode is significantly greater than that of the first infectious event (11.3 years vs. 0.94 years), supporting the notion that the former may be a precondition to the latter [21]. Both CGD-associated colitis and inflammatory pulmonary disease seem to improve post-hematopoietic stem cell transplantation (HSCT), which also suggests a common aetiopathogenic basis [36,37].

However, granulomas which are not directly associated to an infection are also prevalent in CGD, and tissues often fail to grow microbial pathogens in culture [38]. Some authors suggest that, even for those cases, a chronic, undiagnosed (smouldering) infection may explain their formation. Another potential explanation is that the current methods of detecting infectious agents may not be sufficiently accurate.

Other theories propose that the intact components of the immune system, such as T and B cells, may produce an overwhelming response to infection in these patients, with resulting inflammation [39]. Indeed, hypergammaglobulinemia and elevated acute phase reactants are commonly seen in CGD. Cases of very early onset GI and urogenital inflammation in XL-CGD have also been reported, suggesting that inflammation may develop without infection [40]. Importantly, there is a lack of association between NROA and the presence of gastrointestinal symptoms in these patients [41] and female carriers of XL-CGD often exhibit granulomatous complications despite adequate NROA.

Details of why immune hyperactivation ensues are elusive, but a potential explanation is that the absence of ROS in CGD neutrophils may create certain signalling alterations that favour proinflammatory responses. This is because ROS are involved in the regulation of intracellular signalling, e.g., the oxidation of cysteine residues in phosphatases and transcription factors [12]. CGD phagocytes also produce high levels of TNFa and IL8, probably through hyperactivation of NF-kappa B [12].

Pro-inflammatory macrophages secreting IL18 may also contribute to chronic inflammation [42]. Indoleamine 2,3-dioxygenase may perform a significant role in the amplified inflammatory response characteristic of CGD. p47phox knock-out mouse mice that were infected with Aspergillus fumigatus exhibited more damaging inflammatory lung injury than the initial infection as well as demonstrating inefficient tryptophan catabolism as a result of blocked indoleamine 2,3-dioxygenase function [43]. 

The genetic background of individuals with CGD may play a role in determining inflammatory risk. In one study, the IBD genetic risk score, calculated using IBD single nucleotide polymorphism genotype, was higher in CGD patients with colitis than in those without colitis [44]. In p47phox-deficient mice, a particular microbiome signature is also associated with colitis [45]. The intestinal microbiome and metabolomic picture of patients with CGD-associated IBD shows a pattern distinctive from that of CGD patients without IBD and from healthy controls and likely plays a role in determining inflammatory susceptibility [46]. However, it is not clear if an inflammatory milieu encourages a particular microbial and metabolic profile or if the microbes encourage the inflammatory landscape. In a p22phox murine model, disruption of the intestinal mucous barrier potentiated bacterial-induced intestinal inflammation [47], and it is likely that, in some patients at least, similar mechanical barrier disruption is implicated in CGD-associated inflammation.

Defective autophagy may be observed in murine CGD models and human CGD patients and is associated with reactive oxygen species-independent activation of the inflammasome and increased release of IL1β. Blocking the IL1 receptor restores autophagy, decreases inflammasome activation and reduces clinical inflammation in mice and human CGD patients [48].

Attention has also been drawn to the role of apoptosis in these patients [49]. Neutrophils with a defective NADPH oxidase complex are resistant to apoptosis in vitro and produce fewer anti-inflammatory mediators after phagocytosing apoptotic targets [50]. Neutrophils from patients with CGD are also less able to engage the DNA damage–repair process when stimulated and generate more inflammatory cytokines [6]. Although human diseased neutrophils are resistant to apoptosis, nevertheless, in mouse models of XL-CGD, the injection of apoptotic gp91-knockout neutrophils can lead to autoantibody formation [6].

Finally, tolerance mechanisms may be involved. In a murine model, the failure of an immune-modulatory effect of superoxide radicals was associated with exuberant inflammatory responses and Th17-mediated pathology and arthritis [51].

## 6. Treatment

Immunosuppressive agents are often given to treat inflammatory complications in these patients, but their use is hindered by the fact that CGD patients are predisposed to infection. Sulfasalazine or alternative aminosalicylates can be used for mild gastrointestinal disease [4]. Oral corticosteroids are more often given in moderate to severe disease, but their long-term use is linked to multiple side effects, including osteoporosis, suppression of the hypothalamic–pituitary–adrenal axis, diabetes, hyperglycaemia, myopathy, glaucoma, and hypertension. Intravesical corticosteroids are also used to treat urinary complications [6]. Steroid-sparing agents, like azathioprine or sulfasalazine, may offer a solution in this situation.

Realisation of the important role of pro-inflammatory cytokines in the development of inflammatory complications has resulted in the investigation of various cytokine pathways as potential treatment targets. E.g., anti-TNFa monoclonal antibodies have been tried in patients with treatment-resistant or fistulating colitis [52,53]. Infliximab is most commonly used, but this seems to result to an increased risk of infection [52,53]. Adalimumab has also been tried in severe refractory colitis [54]. Evidence on the use of the recombinant IL1 receptor-targeted antagonist anakinra in patients with severe colitis is conflicting, with some authors reporting improvement while others showing marginal or no benefit [55]. The IL23 antagonist ustekinumab has also been trialled. In a cohort of 8 patients with CGD-associated IBD ustekinumab, clinical remission was achieved in half and endoscopic improvement in 6 of those patients. No infections that would lead to discontinuation of therapy were reported [56].

Vedolizumab, a monoclonal antibody that binds the integrin a4b7 heterodimer and blocks its interaction with MAdCAM-1 is another potential option. This prevents leukocyte binding to endothelial surface and its extravasation into inflamed tissue and is proven to be effective in Crohn’s disease [57]. A study of its use in 11 patients showed subjective clinical improvement in 7 and mucosal improvement in more than half, but the response was short-lived [58].

Hematopoietic stem cell transplantation has undergone significant refinements over the years and today is considered to be the standard of care, at least for patients with more severe forms of CGD [59]. All genetic forms of CGD respond to transplantation, and the best outcomes are expected at a young age, before permanent organ damage has occurred [59]. For a few patients where HSCT may not be an option, e.g., because of severe pre-morbid conditions, clinical trials on gene therapy are currently underway [60,61]. However, current methods of introducing the corrected gene generally result in an increased but still sub-optimal oxidase activity, which may be sufficient to protect from infection but not the inflammatory complications previously described.

## 7. Conclusions

Inflammatory complications, particularly those affecting the gastrointestinal, respiratory, and urinary tracts, contribute significantly to morbidity in CGD patients. A high index of clinical suspicion is required to diagnose them, not only in XL-GGD, but also in other forms of the disease. Our understanding of the complex mechanisms that underlie granuloma formation and autoimmune manifestations in these patients is growing, but there are still many questions that remain unanswered. Immunosuppressive treatment options are available, and our therapeutic armamentarium is expanding with the addition of various monoclonal antibodies. HSCT should also be considered in these patients, particularly in those with a more severe form of the disease.

## Figures and Tables

**Table 2:** Inflammatory complications reported in large case series with a majority of AR-CGD patients.

	Rawat et al., 2021 (India) [9]	Fattahi et al., 2011 (Iran) [11]	Mortaz et al., (Iran) [10]	Koker et al., 2013 (Turkey) [8]	Mean Frequency (Range)
**Number of patients**	236 (77 XL, 97 AR)	93 (12 XL, 81 AR)	65 (10 XL, 50 AR)	89 (34 XL, 55 AR)	
**Respiratory ^1^**	16 (7%)	0	23 (35%)	43 (48%)	23% (0–48%)
**Gastrointestinal**	13 (6%)	1 (1%)	12 (18%)	5 (6%)	8% (1–18%)
Gastric ^2^	1	0	5	0	
Colonic ^3^	12	1	7	5	
**Connective tissue ^4^**	2 (0.8%)	5 (5%)	2 (3%)	3 (3%)	3% (0–5%)
**Cutaneous ^5^**	2 (0.8%)	3 (3%)	0	4 (5%)	2% (0–5%)
**Urinary**	1 (0.4%)	0	1 (2%)	0	1% (0–2%)
Renal ^6^	1	0	1	0	
**Cytopenias ^7^**	0	1 (1%)	1 (2%)	1 (1%)	1% (0–2%)
**Hepatic ^8^**	2 (0.8%)	0	1 (2%)	1 (1%)	1% (0–2%)
**Ocular ^9^**	0	1 (1%)	0	1 (1%)	0.5% (0–1%)
**Neuromuscular ^10^**	0	2 (2%)	0	0	0.5% (0–2%)

^1^ interstitial lung disease, granulomas, sarcoidosis, pulmonary nodules, ^2^ gastritis, gastric outlet obstruction, ^3^ ulcerative/Crohn’s /other colitis, autoimmune enteropathy, chronic diarrhoea, ^4^ systemic lupus erythematosus, rheumatoid /reactive/juvenile idiopathic /HLAB27-related /other arthritis, Kawasaki disease, ^5^ discoid lupus, chilblains, lupus-like lesions, ^6^ chronic kidney disease, amyloidosis, ^7^ autoimmune thrombocytopenia, ^8^ autoimmune hepatitis, granulomas, ^9^ chorioretinitis, uveitis, granulomas, retinal vasculitis, ^10^ CNS granulomas.
